# Poisoning accidents in young children—Theory-based evaluation of an mHealth app

**DOI:** 10.1177/20552076251362753

**Published:** 2025-08-06

**Authors:** Patricia Müller, Annett Schulze, Johanna Geppert, Axel Menning, Fabian Brand, Paula Stehr, Doreen Reifegerste, Constanze Rossmann

**Affiliations:** 1 Department Risk Communication, 27652 German Federal Institute for Risk Assessment (BfR), Berlin, Germany; 2 Department Biological Safety, 27652 German Federal Institute for Risk Assessment (BfR), Berlin, Germany; 3 Department of Media and Communication, LMU Munich, Munich, Germany; 4 School of Public Health, Bielefeld University, Bielefeld, Germany

**Keywords:** mHealth, mobile app, usability, evaluation, caregivers, digital health, poisoning

## Abstract

**Background:**

Unintentional childhood injuries such as poisonings are prevalent among young children and can have severe or even fatal consequences. As most of these injuries happen in and around the home, parents and other caregivers need to be prepared to prevent and deal with them. mHealth apps are promising for this, since regular smartphone use is widespread. This study evaluated the German app “Vergiftungsunfälle bei Kindern” (*Poisoning Accidents in Children*).

**Methods:**

Parents (*n* = 21) and temporary caregivers (*n* = 21) of children under the age of seven were recruited for a mixed-methods study, comprising a remote usability test (thinking aloud, observation data and click protocols), focused interviews and the System Usability Scale (SUS).

**Results:**

The mean usability score was 77.6 ± 15.7 out of 100. Most participants reported they trust the app and would use it in specific situations. The general app structure was perceived as clear. Perceived usefulness was facilitated by education on childhood injuries and contact to the poison control center, whereas it was impaired by the perception that the app impedes learning and rapid action in critical situations. Major obstacles included a reduced comprehensibility in terms of text design, visualizations, and complex language, all causing high time expenditure. Moreover, while some participants wished for condensed information, others expressed the need for more detailed explanations.

**Conclusion:**

The results indicate that the intention to use the app was high, but its perceived ease of use and usefulness could be further enhanced by a better tailoring to different needs of caregivers.

## Background

Unintentional injuries such as falls, drowning, burns or poisoning are highly prevalent among young children, most of them occurring in and around the home.^1,2^ As young children usually are curious and tend to put things in their mouth unaware of potential consequences, they are particularly susceptible to poisoning accidents. These can be rather severe or even lead to death because the toxicity of most poisoning agents relates to the dose per kilogram of bodyweight.^3^ Moreover, concerns are raised that the number of poisoning accidents might even rise due to the increased use of concentrated household products (e.g. laundry pods).^4^ The relation between the availability and presence of certain products and childhood poisoning accidents, has been impressively shown during the COVID-19 pandemic: With the expanded use of disinfectants, there was also a marked increase in the reported exposure to them among children.^5^ There is a lack of representative data on poisonings in children in Germany,^6^ but according to hospital diagnosis statistics they are among the most frequent reasons for a hospital admission.^7^ Studies analyzing their prevalence consistently show that unintentional childhood poisonings are a relevant health burden worldwide.^8–11^

However, most of these unintentional childhood injuries can be prevented. There are different approaches for achieving this: (a) engineering, which refers to the modification of products or the environment to make the home safer, (b) enforcement, which is realized through regulation and law-making, and (c) education and skill development.^12,13^ In countries with statutory consumer protection such as Germany, several regulatory measures to reduce the risk of unintentional poisonings in children have already been taken, for example, mandatory child-resistant packaging of potentially dangerous products or the implementation of poison control centers.^3^ For low- and middle-income countries such programs are encouraged as suitable strategies.^13^ These are key factors for prevention, but they neither fully protect against poisoning accidents nor do they relieve parents and other caregivers of their responsibility to ensure safety at home. Therefore, education through providing information, enabling skill development, and raising health literacy remain crucial.^14^ Previous research has highlighted that caregivers tend to underestimate the risk of unintentional injuries and the vulnerability of their children.^15^ In Germany, less than 10% of the parents of children and adolescents under the age of 18 believe their children are at risk in and around their home.^16^ In addition to this, there is evidence that it is difficult for parents and other caregivers to identify hazards, among them poisoning agents.^15^ Moreover, caregivers frequently overestimate children's cognitive skills and have unrealistic expectations of their ability to recognize hazards.^17,18^ Both, not anticipating a risk and having unrealistic expectations of children have been shown to be major contributing factors to unintentional childhood injuries such as poisonings.^18^

These findings underline the pivotal role of educating parents and other caregivers.^19^ Improving their risk perception in terms of unintentional childhood injuries and providing them with relevant knowledge of prevention and handling is beneficial. In this regard, educational mHealth apps are a promising tool. Such apps can be viewed as educational interventions aimed at improving knowledge.^20,21^ As smartphone use is widespread—in Germany over 80% of the general population use mobile Internet at least occasionally^22^—mHealth apps can provide easy and cost-effective access to relevant information. In Germany, the app “Vergiftungsunfälle bei Kindern” (*Poisoning Accidents in Children*) is available. It was developed and published by the German Federal Institute for Risk Assessment in 2013 as a mobile information tool for Poisoning Accidents in Children and their prevention.^23^ This theory-based evaluation study was conducted with the aim to improve this mobile application before a relaunch of an updated version and to thoroughly examine facilitators and barriers of its use.

The current state of research on mHealth apps in the context of childhood injuries is rather scarce. A recent systematic literature review on mobile apps aimed at preventing and handling unintentional injuries in children aged under seven years identified five relevant articles.^24^ Among them, two studies evaluated apps that are both educational in their purpose and encompass a broad variety of potential injuries. The *Grow Up Safely* app provides information on injury prevention tailored to the child's developmental age.^25^ In a similar vein, the *Make Safe Happen* app intends to educate parents and other caregivers on how to make their homes safer for children.^26^ While both studies elicit that parents indeed value the potential usefulness of these apps, they lack a systematic and theory-driven analysis of benefits and barriers associated with their use.^24^ This is a general issue in many evaluations of mHealth apps.^21,27,28^

This study, thus, advances research on facilitators and barriers of educational mHealth app use by making recourse to the *Technology Acceptance Model* (TAM) as a theoretical framework. In particular, it focuses on its two key components determining the behavioral intention to use a technology, perceived ease of use and perceived usefulness. Perceived ease of use refers to an individual's perception that a technology can be used without effort, while perceived usefulness describes the benefits of using it.^29^ Originally developed for explaining IT adoption at the workplace, TAM has been applied in the context of health communication as well,^30,31^ and both perceived ease of use and perceived usefulness have been proven to be significant predictors of the intention to adopt mHealth apps.^32–34^ However, as the majority of TAM studies is quantitative in nature, it largely remains a black box which specific features actually contribute to the perception that a technology is easy to use and useful.^35^ Consequently, the prescriptive guidance for developers and designers is assumed to be rather weak.^36^

Another source of criticism has been the TAM neglecting further potentially relevant factors in the technology adoption process. Therefore, many studies added new variables to the model depending on their specific thematic contexts.^37^ While this can be problematic as research becomes highly heterogenous and reproducibility is hampered,^38^ by this means studies regularly have identified trust and trust-related issues as relevant variables. In terms of trust, it has been shown to be important who provides health information or recommends an mHealth app.^25,39^ Moreover, data security and privacy issues might play a decisive role.^40,41^

The aim of this theory-based evaluation study is twofold. Firstly, we aim to provide theory-driven knowledge on facilitators and barriers of the acceptance of an mHealth app that is designed to convey scientific health information to non-experts. The app *Poisoning Accidents in Children* for preventing and handling unintentional injuries in young children targets parents and other caregivers. Thus, it is an ideal use case. By applying an extended TAM as theoretical framework, we investigate factors that impair or contribute to perceived ease of use and perceived usefulness. Hence, we consider how (potential) user experience characteristics influence these core variables of the TAM. Moreover, we explore how they relate to the behavioral intention to use the app and the role of trust in this. In addition to this, secondly, the study derives practical implications for mHealth app development and improvement.

To take a closer look into the above-mentioned black box and gain in-depth insights, we apply a mixed-methods design with a clear focus on qualitative evaluation measures.

## Methods

### Design

**Figure 1.**
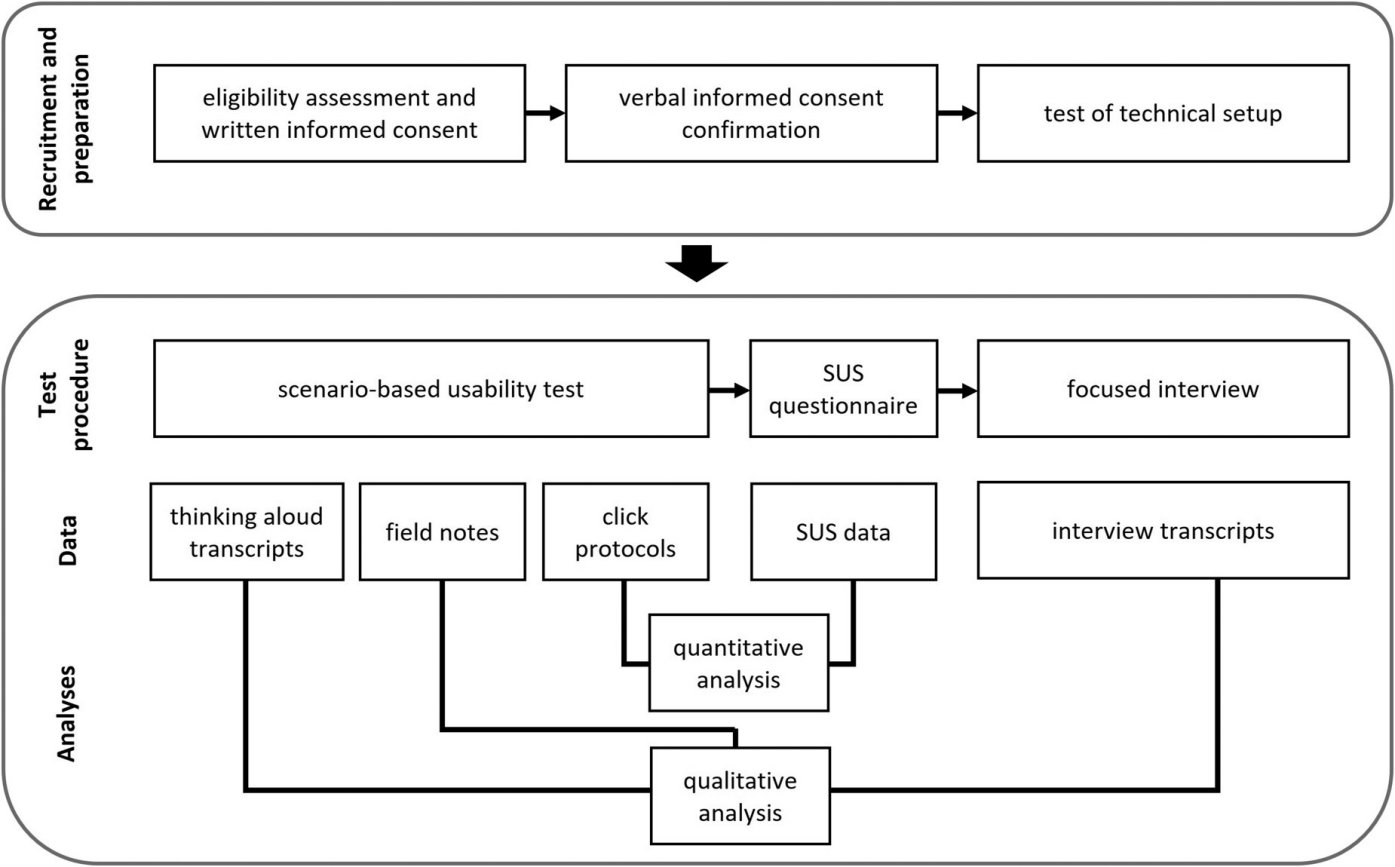
Overview of the study design.

The mixed-methods study combined both qualitative and quantitative measures. The scenario-based usability test comprised thinking aloud and observational data (click protocols and field notes). It was followed by a short questionnaire using a slightly modified version of the System Usability Scale (SUS)^42^ and a focused interview (see Figure 1 for an overview). As the main analytical focus of the study was on the thinking aloud protocols collected during the remote usability test and the subsequent qualitative interviews, we used the Consolidated Criteria for Reporting Qualitative Research checklist as a guide for preparing the article (Supplemental Appendix S1).^43^

The members of the research team that were engaged in the data collection and data analysis had different professional backgrounds (i.e. communication science, sociology) and degrees (i.e. MA, PhD). Albeit, all of them are experienced in qualitative and quantitative methods. Ethical approval was granted by the Ethics Committee of the University of Erfurt, Germany (No. 20210512).

### App “Poisoning Accidents in Children”

The app *Poising Accidents in Children* can be installed on Android as well as iOS devices and, in terms of data protection, it does only require system privileges and no input of any personal data. In general, the app's purpose is to inform parents and other caregivers of young children about poisoning risks that occur in the household, with plants, or with medication. Over-the-counter and prescription medications such as paracetamol, antidepressants or analgesics, household products such as bleach, detergents or disinfectants and poisonous plants are the most common agents involved in childhood poisoning.^3^ In addition to information on numerous toxic agents, the app includes general advice sections on prevention (i.e. how to equip a medicine cabinet) and first aid measures (e.g. for burns or swallowing).

**Figure 2.**
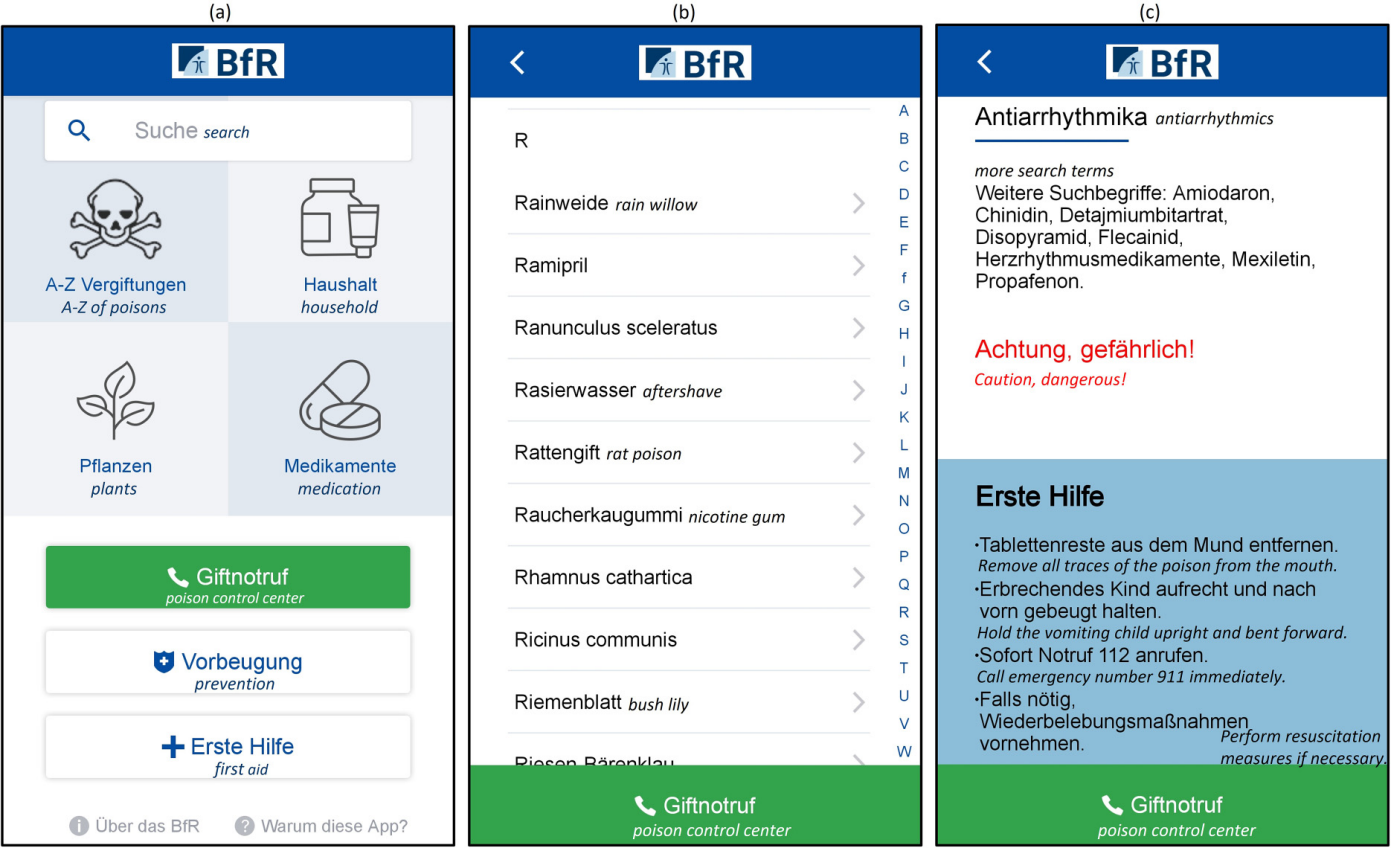
Selected screenshots of the app: (a) start screen, (b) alphabetical list of potential causes of poisoning, and (c) subpage with first aid information on antiarrhythmics. *Note*: English translations added for clarification.

On the app start screen users find four main areas (Figure 2(a)): They can view an A–Z list of possible poisons (Figure 2(b)) or find out more about poisonings in the household, caused by plants, or by medication. An integrated search function allows to specifically search for substances, products, plants, or medication. The respective subpages (Figure 2(c)) usually contain an overview of further search terms, an info-box with instructions for first aid, information on ingredients, and the poisoning pattern. Depending on the poisoning agent, there might also be highlighted warnings, information on when a pediatrician should be contacted, and/or tips on preventing the poisoning. In case of an emergency, the app offers the possibility to call the poison control center (PCC) via an integrated button that is permanently displayed. This also important against the background that the app does not aim to replace counseling by qualified medical specialists.

The usability test was conducted with the app version available in the app stores at the time of the study. No modifications have been made specifically for research purposes.

### Participant selection

Participants were eligible for the study, if they were either parents of children under the age of 7 years or informal, temporary caregivers such as babysitters, neighbors or family members that do not live in the same household as the child they take care of. Both are main target groups of the app, as most unintentional poisonings occur at home.^1^ Furthermore, possessing a smartphone and an audio-capable desktop PC or notebook was required for inclusion in the study.

Parents or temporary caregivers of children aged seven or above were excluded. Further exclusion criteria for participation were:

- only taking care of children under the age of 7 years in a professional position (e.g. as a kindergarten teacher)
- currently working or having worked in the advertising industry, market research, marketing, as a product manager or UX/UI designer
- only possessing a tablet PC but no smartphone and audio-capable PC or notebook
- not using smartphone apps in general.

A major challenge in developing an educational mHealth app is that it has to match the needs of an audience with varying communication competencies as well as levels of health literacy.^44^ Beyond these eligibility criteria the sample of a usability study hence should cover varying types of potential users. Thus, we applied a purposeful quota sampling strategy. It aimed for an even distribution of gender and caregivers with a diverging attitude towards mHealth apps (open/positive or skeptical/negative). Accordingly, further variables such as demographics and the experience of the participants with mHealth apps were assessed.

The recruitment process was realized in cooperation with the professional German market research institute “eye square.” They were responsible for recruiting a target sample of at least twenty participants in each caregiver group throughout Germany. While it is partially argued that five participants are sufficient in usability testing, empirical evidence shows that using a sample size of minimum twenty is more reliable (i.e. 95% of usability problems are detected).^45^ Moreover, we aimed for this number of participants to be able to detect a variety of perspectives in the qualitative interviews. The sampling strategy allowed to reach data saturation.^46^

For the recruitment the market research institute relied on their existing panel of potential test persons. By using a screening questionnaire, individuals who met the eligibility criteria were detected. Informed written consent was obtained during this process and prior to participating in the study. All participants received an appropriate expense allowance.

### Data collection

The data collection took place in September and October 2021. As this was still during the COVID-19 pandemic with several restrictions present, the usability test was conducted remotely by using the videoconferencing software Zoom. This ensured the best possible health protection for the participants as well as the researchers and had several methodological advantages: Participants could be recruited Germany-wide, they could do the test with a familiar device in their home environment, and the field observation was rather unobtrusive as the researchers were not visible.

Before the remote usability test was set up, participants were prepared by an employee of the market research institute: As part of a technical consultation, all participants received instructions on how to install and configure the app, were introduced to the web conference system and familiarized with the card sorting task which was one part of the remote usability test. Prior to the study, participants had no contact to the researchers responsible for the data collection. Before the test, however, they were informed about their affiliation—all were employees of the German Federal Institute for Risk Assessment—and the reasons for the app evaluation.

The remote usability test proceeded as follows. After a brief introduction into the study and a second, verbal, confirmation of informed consent to participate, the scenario-based test session was conducted. It was moderated by one of two male members of the research team (FB, AM). After completing all test scenarios, respondents were asked to fill out the modified SUS questionnaire before the study concluded with the focused interview.

In order to retrace the participants’ individual task processing, they shared their smartphone screen with the research team during the whole remote usability test but not during the qualitative interview. Furthermore, all remote usability tests were observed and field notes were taken on how successful the participants completed the tasks, how they navigated through the app and on remarkable reactions such as moments of frustration, nervousness or joy. This observation was always done by one of the female researchers who also conducted the subsequent interview (JG, AS), which allowed them to explicitely refer to the previous remote usability test. All individual test sessions including the focused interviews were audio-recorded.

#### Test scenarios

The remote usability test comprised seven scenario-based tasks the participants had to process by using the app. They covered a broad variety of hypothetical but realistic use cases, both in terms of prevention and handling of emergency situations (Table 1).

**Table 1.** Overview of the test scenarios.

	Task—Please use the app to…	Task fulfilled when…
1	*childproof your home.* Participants should imagine that they have moved to a new apartment and ask themselves: How can poisoning accidents with children in the home be prevented?	*participants find relevant information.* While there is a specific prevention section, it was also sufficient if participants found relevant information in other app sections.
2	*assess the risks of eight different products.* Participants should imagine they want to find out something about the potential risks of cosmetics and products in their bathroom for young children (e.g. soap).	*participants find the product.* It was required that the participants find the product, but they had not to evaluate the risk completely correct necessarily.
3	*check whether a plant is poisonous.* Participants were shown three pictures of the poisonous plant *giant bear claw*.	*participants find the plant.* It was required that participants find the plant and say that it is poisonous.
4	*find out what to do in case of a swallowed paperclip.* Participants should imagine that their child has swallowed a paperclip and ask themselves how to react best in such a situation.	*participants find first aid information.* It was required that participants find information on first aid in case of a child swallowing a foreign object.
5	*check if medicine cabinet is well-equipped.* Participants should find out if they have everything needed for an emergency in their medicine cabinet.	*participants find relevant information.* It was required that participants either find the checklist in the first aid section or the tips given in the prevention section.
6	*find out what to do in case of a swallowed tablet of *Ramipril.** Participants should imagine that their child has swallowed a tablet of *Ramipril*, an ACE inhibitor, and find out how to react.	*participants find out what is recommended in this situation.* It was required that participants find information on the medication and name measures.
7	*contact the poison control center (PCC).* Participants should imagine that their child has cleaning agent residues all over the face and call the local poison control center.	*participants identify the PCC to be called.* It was required that participants choose the responsible poison control center either from the list or via GPS tracking.

All scenarios were intended to reflect specific information needs in the context of childhood injuries and varied in terms of the presumed difficulty. Further criteria were, that the tasks had to take into account different places in and around the home where (poisoning) accidents could happen (e.g. the bathroom, the garden), and they had to target the use of various sections and functionalities in the app.

While navigating through the app, the participants were encouraged to express their thoughts and task processing (thinking aloud). They could interrupt a task at any time, if they were not able to come to a proper solution by using the app or for any other reason. The remote usability test and the subsequent completion of the SUS took an average of 49 minutes with a range between 30 minutes and 1 hour and 16 minutes.

#### Interview guide

For the qualitative interviews a semi-structured interview guide was developed and pilot tested (AS; Supplemental Appendix 2). It aimed to capture core dimensions of the TAM as well as extend its scope by focusing on the user experience (e.g. in terms of content, navigation, and design), on recommendations for optimizing the app and on trust-related issues. Thus, the interview guide was structured in four different thematic blocks, each of which consisted of a set of fixed questions but also allowed for follow-up questions. (a) In the first one, perceived usefulness of the app was captured, and ascertained in which use scenarios the app was more or less helpful as well as reasons for that. (b) The second block concentrated on perceived ease of use. Participants were asked to reflect on barriers in using the app, but also on aspects which facilitated finding needed information. (c) Following this, it was discussed how the app could be improved. (d) The fourth block comprised questions on trust, such as trusting the app for advice in specific situations (i.e. prevention, critical injury situations), the role of the app publisher, privacy, and data security. The interview concluded with a question on participants’ willingness to keep the app installed on their smartphone and the opportunity to address further issues relevant to them.

The duration of the interviews was on average 25 minutes and varied between 15 and 40 minutes.

### Data analysis

All audio recordings were transcribed verbatim by a professional German transcriber. At the same time identifying information was removed from the transcripts. For the qualitative coding we used the analysis software MAXQDA 2020. Based on the deductive categories derived from theory, that had already structured the thematic blocks of the interview guide, a rough coding scheme was developed and applied (PM, AS). Afterwards, these categories were refined inductively by systematically going through the material again, following the approach suggested by Mayring.^47^ This analysis step was done by PM. Additionally, the codes were reviewed by AS, another experienced researcher in qualitative research. At last, in another coding round all codes were checked again to ensure that they were correctly assigned to the sub-categories defined in the final coding scheme (PM).

As the interviews were conducted in German, all quotes presented in the “Results” section were translated by the first author.

## Results

### Study participants

In total, 42 individuals took part in the remote usability test (Table 2). Five participants that had originally signed up for participation canceled or did not show up without giving a specific reason. Twenty-one permanent caregivers, that is, parents, and 21 temporary caregivers, such as non-professional babysitters and neighbors, participated. Among all, only five participants had never used an mHealth app before. The mean age of the participants was 39 years with the youngest being 19 and the oldest 65 years old. As expected, participants in the group of the permanent caregivers were more homogeneous in terms of age. Moreover, the distribution of education was slightly skewed towards higher education in the group of parents. Considering the attitude towards mHealth, male temporary caregivers more often showed a positive (*n* = 9) than negative (*n* = 2) one.

**Table 2.** Overview of the sample.

		Permanent caregivers (*n* = 21)	Temporary caregivers (*n* = 21)
Gender	Female	10	10
	Male	11	11
Age	18–35 years	8	8
	36–50 years	13	5
	51+ years	-	8
Education	Middle education and lower	6	10
	Higher education	15	11
Attitude toward mHealth	Positive	11	13
	Negative	10	8

### Task performance and overall app usability

On average, participants completed 84% of the tasks (*n* = 14, seven test scenarios with scenario 2 comprising eight separate tasks, cf. Table 1). The percentage of tasks completed varied between 36% and 100%. Only one test person was able to reach the highest score. All participants identified the correct PCC to call (100%, scenario 7) and almost everybody detected the first aid information on the swallowed medication Ramipril (98%, scenario 6). Furthermore, the majority of the participants managed to find out what to do if a child has swallowed a paperclip (88%, scenario 4), what a medicine cabinet should contain (83%, scenario 5), and how to childproof the home (81%, scenario 1).

Clear patterns emerged as to which tasks were the most demanding. All but one participant could not find information on *giant bear claw* in the app (scenario 3), as only pictures of the plant were shown in the task description. Thus, participants had to know the plant to solve the task, which made it extraordinarily difficult. Some gave up rather quickly after one click, others searched several sections and different listed plants making up to 36 clicks. Further difficulties were observed in the second scenario. While the participants were able to find the products for the risk assessment in most cases (88–98%), it posed a challenge to detect information on *Baldriparan* (55% success rate), which is a trade name for valerian (German: Baldrian) in Germany.

The mean total SUS score was 77.6 (SD = 15.7) out of 100, indicating good but not excellent usability.^42^ While nearly a third of the participants (*n* = 13) rated the usability as excellent or even best imaginable, the score of three test persons showed it was perceived as awful. Participants who evaluated the app more positive, meaning their score was at least signifying a good app usability, tended to perform better (task completion: *M* = 12.1, SD = 1.1, *n* = 30) than those who rated the usability of the app as just ok or even awful (task completion: *M* = 10.8, SD = 2.5, *t*(13) = -1.81, *p*-value = 0.095, *n* = 12).

### Results of the theory-driven qualitative analysis

**Figure 3.**
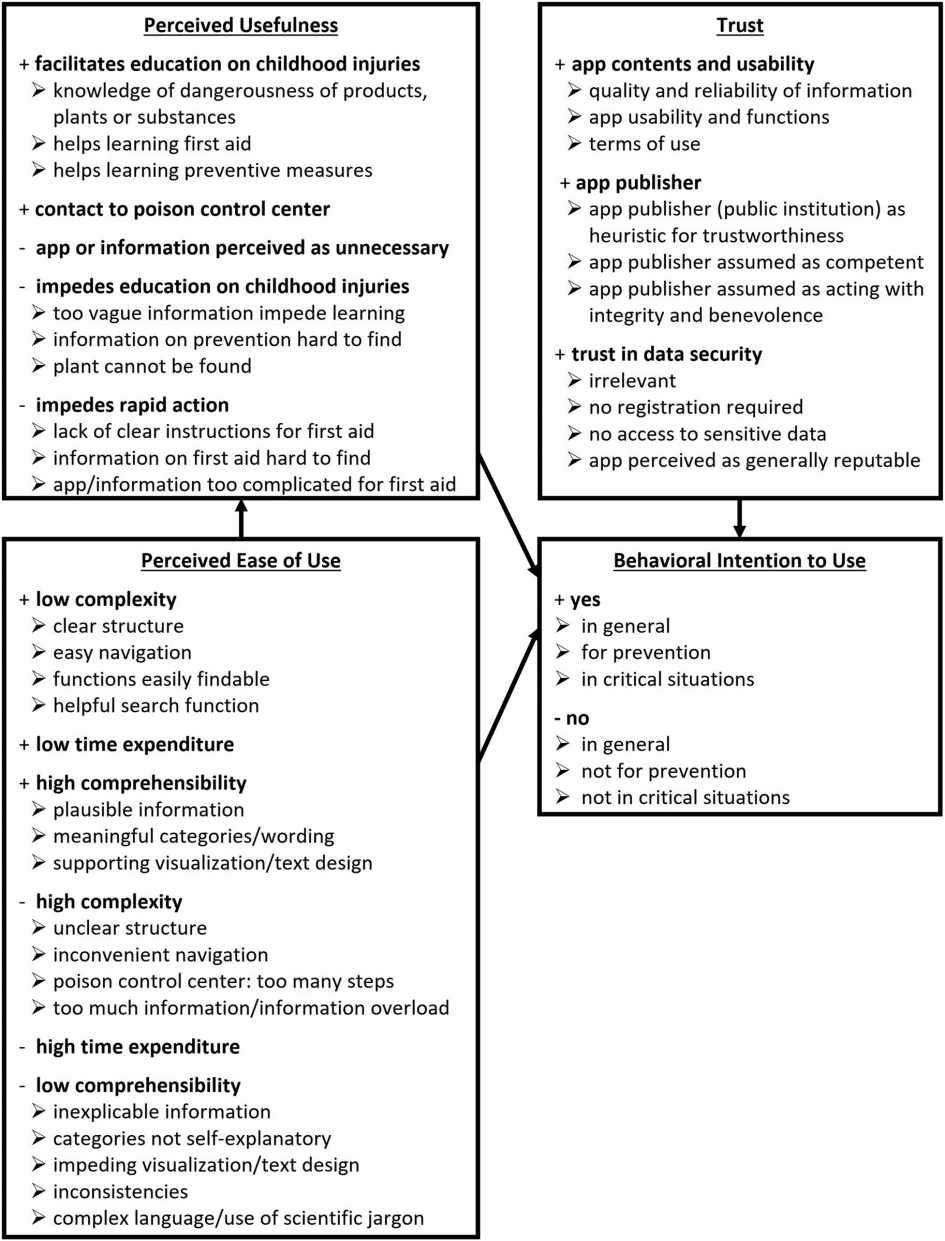
Overview of the main and sub-categories of the qualitative study; research model based on TAM.^29^ *Note*: The + signs and – signs indicate a positive or negative perception.

The results of the qualitative parts of the study are structured according to the extended TAM model with trust added as an additional variable (Figure 3).

#### Perceived usefulness

Perceived usefulness refers to the assessment of how beneficial using a technology is.^29^ According to our results, it comprises two main categories in favor of the app: the facilitation of education on childhood injuries and the contact to the PCC.

Respondents valued that the app conveys knowledge on both preventive measures and first aid. Although caregivers were aware of some typical hazards, the app encouraged reflecting on prevention and be more conscious about the matter, as one participant described:

“Yes, I have some cleaning products under the sink, I have descaling agent under the sink, that has to go, and indeed quickly, and I’m also fully aware that medication should not be near children. I have a few things by my bed because I need them by my bed every day, but they also need to get away from there. That is also a realization. They go in the hanging cupboard in the bathroom. Children can’t get to it.” (male temporary caregiver, 65 years old)

Moreover, interviewees claimed that they perceived the app as useful because they learned about the dangerousness of different products, substances, or plants. In this context, they were often surprised that they had not correctly assessed the risks of specific products such as nail polish or baby powder, as one respondent noted:

“I don’t use it, I don’t know the ingredients of nail polish either, but I would have thought it was more dangerous. With baby powder I also thought, ‘oh what's the baby powder going to do – what's going to happen?’. But of course, you can inhale it and it goes to your lungs. That's when you trivialize things.” (father, 36 years old)

In terms of education about first aid, especially parents and participants with a positive attitude towards mHealth referred to the usefulness of the app for enabling coping with critical situations. The app did not only provide new information, but also helped to refresh existing knowledge:

“I already knew from a first aid course for children back then, but that's exactly what was recommended there, but it's also written here completely accurate. So yes, now I know what I need to do.” (father, 39 years old)

According to the interviews, the possibility to call the PCC was considered a valuable option, as it enables personal advice from medical professionals in an emergency.

The aspects hampering perceived usefulness were summarized in three categories, namely, that the app or the information conveyed are perceived as unnecessary, that it impedes education on childhood injuries and rapid action in an emergency. Very few respondents (*n* = 5) stated that they perceived the app or certain information as useless at all.

A common theme was that participants criticized not being able to find a plant without knowing its name. The corresponding task (scenario 3) could hardly be resolved. In this regard, the participants saw a huge potential for optimizing the app, be it by rather simple measures such as adding a picture to every plant name in the alphabetical index or more advanced ones such as integrating an image recognition software or photo scanner:

“So, I would like to see a camera symbol, for example, so that you can open the camera and take a photo of the plant and then the app will pick out the right plant species.” (female temporary caregiver, 30 years old)

Beyond that, both vague and hard-to-find information on prevention impeded learning about childhood injuries. Assessing how dangerous a hazard actually is posed a specific challenge to some respondents. Others expressed the impediment of rapid action in a critical injury situation:

“But if I’m in a situation where my child has somehow just emptied ‘Domestos’ [an all-purpose cleaner] I want to see it straight away: Is it bad, so I’m right to panic, or do I continue to watch the show I’ve just watched or read my book?” (father, 51 years old)

Reasons given by the participants were the lack of clear instructions for first aid and that relevant information were either hard to find or too complicated to act on. A further related point of criticism was that the app does not immediately dial the PCC. Instead, users have to click through another page with preparatory questions and then either select their region or city or use the GPS localization function.

#### Perceived ease of use

As suggested in the TAM, perceived ease of use not only can influence the behavioral intention to use a technological innovation such as an app but also impacts its perceived usefulness.^29^ Thus, a majority of aspects contributing to or impairing perceived ease of use can be directly linked to it. We derived three main categories: complexity in terms of structure and contents, time expenditure and comprehensibility of information and visualizations.

In general, caregivers emphasized that the app was easy to navigate in most cases:

“As I said, the app is much more than user-friendly, even for people who are not so tech-savvy and use their smartphones a lot. So, I think it's very good.” (female temporary caregiver, 57 years old)

This is further supported by the notion of a clear structure, in particular, due to the alphabetical list of potential poisons and the start screen with only a few categories. In terms of high comprehensibility, the corresponding visualizations, that is, symbols for each category and highlighting of important facts in color, were predominantly perceived as unambiguous and beneficial (cf. Figure 2(a) and (c)). Due to its visual presence, moreover, the function for calling the PCC could be found effortlessly. Overall, supporting visualizations and text design as well as the plausibility of information appeared to be crucial for a positive evaluation of comprehensibility. Compared to rather skeptical participants, interviewees with a positive attitude towards mHealth apps also explicitly mentioned that the categories in the app are named meaningfully. All these aspects summarized contributed to the perception of a low time expenditure, which was in addition fostered by the search function. Tasks during the usability test involving it, such as searching for *Ramipril* (scenario 6), yielded the best results in terms of task performance and were perceived as easily solvable. In particular, younger and test persons with a more positive attitude towards mHealth apps expressed that they could find information quickly.

However, we could also identify several factors that impaired the perceived ease of use of the app. First and foremost, the interviewed caregivers criticized that visualizations and text design at times were not comprehensible enough. This was primarily attributed to the poor structuring of texts (e.g. the lack of organizing bullet points) and inconsistencies (e.g. the poisoning pattern or information on first aid occasionally missing). Complex language and scientific jargon posed a further challenge. Besides the general avoidance of mere Latin terms for plants, specific terms such as aspiration or medical charcoal would need more clarification.

The need for more clarity was also discussed in terms of the naming of particular categories such as “prevention,” which at times were deemed as too broad, and information that are not self-explanatory. To be able to grasp decisive information, participants, for instance, wished for a better differentiation of tolerable versus harmful exposure levels to substances, at best illustrated by intuitive visualizations such as a traffic light scheme, a scale, or colored symbols, and for better guidance:

“Decongestant drops, when should I give the child decongestant drops for which things, which of the toxins causes bloating? Or medical charcoal, when should I give the child medical charcoal? In case of which substances?” (female temporary caregiver, 52 years old)

Our qualitative data indicated that information in the app were related to caregiver's own assumptions, and partially, even questioned by some interviewees, underlining the need for more detailed explanations in the app. At the same time, other participants said that the app contained too extensive explanations, which made it complex and could lead to feeling overwhelmed and distracted, especially in a critical situation, as described by a caregiver:

“Because it's just a lot of, lot of, lot of text and I don’t think you want to read a long text in a dangerous situation, you want to get to the information you need as quickly as possible.” (male temporary caregiver, 25 years old)

In some sections of the app, its structure led to confusion. In particular, participants criticized that searching for a specific product, for example, soap, at times resulted in getting shown an app page that simply referred to another one (in this case: all-purpose cleaner) instead of immediately presenting relevant information:

“Then I think, hmm, can’t it say the same thing so I don’t have to go to all-purpose cleaner again.” (mother, 31 years old)

While it would be beneficial to find relevant information where expected, some respondents also encouraged to at least insert links. According to the interviews, both measures would reduce the perception that the app is too time-consuming. Another issue raised, was that it took too long to call the PCC due to the required steps, which also contributed to a high amount of time spent. In particular, participants more skeptical towards mHealth apps criticized this.

#### Trust

As previously theorized, trust is a further potential factor for the behavioral intention to use an app. In our study, most notably, all participants expressed that they trusted the app. However, ambivalent feelings in a critical situation were discussed at times, as described by an interviewee:

“But of course, I wouldn’t trust it [the app] completely and do without professional staff or anything like that. If I felt very insecure, I would still call an ambulance.”

(female temporary caregiver, 32 years old)

Looking more closely at what determined trust, three main categories were identified. It was judged on the basis of app contents and usability, the app publisher, and in terms of data security. Some of the respondents stated that the apparent high quality and reliability of the information offered or the compelling app usability and design influenced their perception that the app is trustworthy. Terms of use, in general, played a minor role, but the app being ad-free and fully disclosing who is responsible for the contents appeared to be trust-enhancing.

A noticeable cue for trust was the app publisher, a public authority:

“Well, it is made by the BfR [German Federal Institute for Risk Assessment], I think it's a reliable source.” (male temporary caregiver, 31 years old)

While the German Federal Institute for Risk Assessment served as a heuristic for some interviewees, others substantiated their trust assessment in greater detail by addressing the publisher's assumed competence or integrity and benevolence. The latter was reflected in statements stressing that there seemed to be no intention to generate profits as the app is free of charge, and that a public authority is perceived as acting independently and for the good of society.

Data protection and privacy are specific aspects of trust in mHealth apps.^40^ None of the participants stated that they were concerned in this regard. For some, however, it was just irrelevant. According to the interviews specific assets of the app were that no registration is needed and it does not require the disclosure of personal data:

“Yes, I wasn’t asked to register in any way, that I had to create a profile or anything. You usually have to do that or you have to pay for it. It's an information app and I found it very secure.” (mother, 33 years old)

#### Behavioral intention to use the app

Almost all participants (*n* = 39) claimed that they would keep the app installed after the remote usability test. In accordance with this, a variety of reasons for the behavioral intention to use the app were discussed: the possibility to look up information in critical situations, to inform oneself about prevention in general, about the dangerousness of products and substances or plants, and the option of contacting the PCC. Perceived barriers such as dissatisfaction with the usability rarely led to the intention to delete the app.

The qualitative data, however, point to the pivotal role of both situational factors and perceived health competencies in considering the use of an educational mHealth app. Due to an assumed time loss and stress reaction some participants would hesitate to use it in a critical situation. In particular, temporary caregivers were reluctant to use the app in an emergency. In cases that appear to be less threatening, however, accessing the app could be an option, as one participant described:

“I can imagine using the app when I’m just so unsure. But more in a less ‘dicey’ emergency, not an acute situation.” (father, 37 years old)

Other respondents would not use the app in terms of prevention, because they do not see the need. Among them, there were more male than female caregivers. Some participants explained, they already feel sufficiently prepared, argued that nothing had ever happened, or simply referred to common sense. Others would rather look up information online than use the app.

## Discussion

### Main findings

Unintentional childhood injuries such as poisonings can represent a serious burden–not only to children and their families, but also economically. Most, however, are predictable and preventable,^1^ with one key factor being the appropriate education of caregivers. In this context, mHealth apps seem to be promising, as they gained importance in health communication over the past years.^48^ This mixed-methods study evaluated an educational mHealth app specifically designed for preventing and handling of poisoning accidents among young children. Thereby, we relied on TAM as a well-established theoretical framework with the aim to gain theory-based insights on facilitators and barriers of the acceptance of an educational mHealth app.^49^ Our results indicate that parents and other caregivers overall were open-minded towards using the studied app and, in general, would trust it. This is in line with previous research on mHealth apps for the prevention of unintentional childhood injuries.^25,50^ However, the findings also revealed a series of barriers in terms of perceived ease of use and perceived usefulness that need to be addressed.

The quantitative analysis of the SUS data showed an acceptable usability and suggested a positive correlation between the SUS rating and task fulfilment rate. This is not surprising as difficulties while performing the scenario-based testing might have inclined a rather negative reflection on the app, particularly on items related to ease of use (e.g. “I thought the app was easy to use,” “I found the app unnecessarily complex”).^51^ While the SUS can provide a good first impression, it remains unknown which actual app features determined this assessment.^42^ Thus, with our qualitative results we shed more light into this “black box,” that is also a common source for criticism in TAM studies.^35^ By relying on the thinking aloud protocols and subsequent interviews, we could both confirm the well-proven relationship between perceived ease of use, perceived usefulness and behavioral intention to use as well as the role of trust,^31,52,53^ and identify factors specifying these central constructs in the context of an educational mHealth app. In this regard, the impressions and needs of potential users varied in several ways, which leads to the conclusion that there is no “one-size-fits-all” app.

Our qualitative results highlight, that three sub-dimensions–complexity, comprehensibility, and time expenditure–shaped the ease-of-use-perception (cf. Figure 3), and are crucial in the evaluation of educational mHealth apps. General critical points were the missing of internal links between app sections, which made navigation inconvenient, as well as long and tedious to read text passages without structuring elements such as bullet points. As time is critical in cases of poisonings,^3^ the associated high time expenditure appeared to be a barrier to using the app, especially in critical situations, which is in line with a qualitative study on an app for acute childhood illness.^54^ Significant facilitating factors were a clear structure and comprehensible visualizations. This finding aligns with prior research also emphasizing the importance of an easy-to-understand interface design.^25,37,55^

Considering the diversity of potential users, our results point to a fine line between a perceived information overload and the need of some caregivers for more detailed information. This is in accordance with the results of a recent qualitative study analyzing publicly available user reviews sampled from mHealth apps providing information for caregivers: While some commentators wished for simplification, others appreciated detailed information.^56^ Moreover, assessments of whether information was plausible or difficult to understand, differed. Both is likely to be attributed to varying prerequisites in terms of information and health literacy.^44^ Health literacy, thus, should not only be considered as an outcome of receiving healthcare information, for instance, provided by an app, as in previous studies.^57^ We argue for a reflection on the role of literacy and diverging user needs already during app development.^58^ Acquiring adequate knowledge by, for example, using an mHealth app, should not only be a responsibility of the individual. Rather, creators of such tools, such as public health institutions, should strive to ensure that they are inclusive for all members of their target group. This implies a more closely review of comprehensibility, in terms of plain language and text design, but also tailoring of information. Previous research has already highlighted the tailoring of content as one central asset of mHealth apps.^44^ In terms of preventing and handling unintentional injuries, tailoring could, for example, accommodate the need of caregivers for information in relation to child age and development.^59,60^ Our results indicated that the app *Poisoning Accidents in Children* does not sufficiently make use of this advantage, perhaps because it was developed based on an information brochure. This is not only reflected in terms of ease of use but also in the barriers discussed with regard to perceived usefulness. All of them are associated with either information being not comprehensible enough or hard to find.

Perceived ease of use and usefulness in interaction with diverging user needs and prerequisites, moreover, determined conceivable use scenarios. While some caregivers would consider the app both for preventive purposes and in an emergency, others referred only to one of these situations. The observation that some participants stressed that they do not need an app for prevention because they already felt well-prepared and did not encounter any emergency situations before, could indicate that the susceptibility of toddlers and preschool children to unintentional injuries, such as poisoning accidents, is underestimated, as already shown in previous studies.^15^ Prevention was primarily emphasized by female caregivers. Since the related tasks almost all referred to the domestic environment (e.g. childproof the home), though speculative, this might suggest that women, especially mothers, still feel more in charge for the safety of their own household and, thus, have a higher information need.^61^ Another explanation could simply be that men prefer other information channels. With regard to critical situations, some caregivers in particular valued the integrated function to call the PCC. This corresponds to previous research that showed that many parents feel the need to seek a professional opinion in a critical situation.^54,62^ However, this preference for a professional assessment can also lead to not trusting the app in an emergency at all, which–as previous research pointed out–might also be related to differing levels of health literacy.^57^

In general, trust in the app was high. This perception was primarily based on either the app contents and usability or the app publisher. The latter is in line with previous studies, that showed that the acceptance of mHealth apps is fostered when they are published or recommended by official sources such as health authorities.^25,63,64^ They can either serve as a heuristic^65^ or are associated with known components of trustworthiness, that is, competence, integrity, and benevolence^66^—both was reflected in our results. Trust in data security was appreciated by caregivers, however, it was not decisive. As the studied app mainly serves an informational purpose, it does not require a registration or access to sensitive data. Therefore, unlike with regard to mHealth apps on, for example, diabetes, cardiovascular diseases, or mental health,^40^ data-related issues were no obstacle.

Based on the results, this study calls for several app improvements in terms of how information is presented. At first, it would be an advancement to begin every text with the most important facts in plain language.^67^ Comprehensibility has been consistently shown to be crucial in mHealth apps across various health contexts.^68,69^ Visualizations, such as a traffic light system immediately signalizing how dangerous a substance or product is and step-by-step instructions for first aid, were thought to enhance the perceived ease of use. In general, precise information on first aid measures would accommodate the identified needs of caregivers. Moreover, if it is not necessary to take action, this should also always be clarified as respondents expected information on how to proceed. Ergo, all texts should be structured in a consistent manner. For those of the caregivers who are keen on additional information, more detailed explanations could follow without lessening the user experience for those who tend to be overwhelmed by too much text. In order to overcome language barriers, jargon should be removed as much as possible.^70^ The integration of new functions such as a photo scanner for poisonous plants could further benefit the app, as also suggested in another study analyzing the mobile health behavior of caregivers.^71^ To sum up, it is essential to make more use of the possibilities a mobile app offers in terms of functionalities and design. First steps in improving the app have already been taken (e.g. improved first aid section). Other modifications such as the inclusion of more links in texts and refinement of the content are still due.

Despite the growing popularity of mHealth apps, research on mHealth apps targeting the prevention of unintentional childhood injuries and safety behaviors is scarce.^24,72^ Our study contributes to this existing body of knowledge. In sum, our results highlight that educational mHealth apps such as *Poisoning Accidents in Children* are well-received by most caregivers and, therefore, can be an additional tool within the range of measures already taken to prevent unintentional childhood injuries, such as mandatory child-resistant packaging.^3^ Appropriate education of parents and other caregivers can help to reduce poisoning rates^19^ and the risk of experiencing repeated poisonings in children.^73^ Therefore, it is crucial to not only develop suitable educational materials but also to ensure and improve their accessibility.^74^ In the German primary healthcare system, a dense network of pediatricians and general practitioners as well as regular medical examinations for children anchored in legal guidelines ensure close monitoring of a child's development and health status.^75,76^ As previous research has shown, health practitioners are one important gateway for information on childhood injury risks and safety behaviors,^77–79^ and they could also be utilized to effectively raise more awareness for mHealth apps such as *Poisoning Accidents in Children.*

### Limitations and future work

This study has a number of strengths: The mixed-methods design gave in-depth insights into facilitators and barriers for using an educational app. In health communication there seems to be no optimal TAM version.^80^ However, perceived ease of use, perceived usefulness, and trust have repeatedly been shown to be key variables,^31^ and our approach relying on a diverse sampling of both parents and temporary caregivers allowed to characterize them comprehensively, taking into account different needs and perspectives.

Nevertheless, there are some limitations. Due to the COVID-19 pandemic, all test sessions were conducted remotely. This more natural testing situation, in the home environment and by using the own smartphone, seemed to be beneficial in terms of both external validity and effort required by the participants. In this setting privacy can be an issue and prevent participants from sharing personal information.^81^ In our usability study, though, this was not required. Moreover, technical equipment is needed,^81^ which might have excluded parents and temporary caregivers that are less tech-savvy. With regard to the test situation, much less control during the usability test posed a challenge. It was more difficult for observers to assess a test person's reaction, for example, if they are just diverted or are actually experiencing difficulties. We also cannot fully rule out that our results were influenced in favor of the app by the fact that the study was conducted by employees of the German Federal Institute for Risk Assessment as sponsorship effects are common.^82^

In terms of the scope of the study, albeit using a real app, due to the artificial test situation we can neither derive conclusions about patterns of app use over a longer period of time nor on how caregivers might benefit with regard to gained self-efficacy and knowledge on prevention and handling of childhood (poisoning) accidents. It would be interesting for future studies to integrate this longitudinal perspective. This would also shed more light on the connection between the behavioral intention to use an app and its actual use, as intention does not automatically result in behavior, even if an app or a specific function was evaluated positively.^26^

From a theoretical point of view, our extended TAM might not have considered all relevant factors. In particular, personal characteristics such as health literacy should receive more attention. Perceived ease of use can be a starting point to more thoroughly address questions of complexity and comprehensibility from the perspective of health literacy. Therefore, the target group of an mHealth app should ideally be involved in its development (*user-centered design approach*).^24^ A more user-centered app development would also enable better tailoring of such mHealth tools which may increase equal access to and more inclusive use of them.^83^

## Conclusions

Educational mHealth apps such as *Poisoning Accidents in Children* can be a viable source for parents and other caregivers in terms of preventing and handling unintentional childhood injuries. With our study being one of the few to date that provides theory-driven knowledge on barriers and facilitators of the acceptance of an mHealth app that focuses on conveying scientific health information to non-experts such as parents and other caregivers in private settings, we contribute not only to the state of research but also provide practical information for the development and improvement of such apps. Our results highlight that there is no “one-size-fits-all” solution that suits all users equally when it comes to perceived ease of use and perceived usefulness. Rather, it is vital to tailor an mHealth app to diverging needs and expectations of potential users. However, comprehensible visualizations, well-structured texts in plain language, and an intuitive navigation that allows using an app without high time expenditure can be assumed as fundamental.

## Supplemental Material

sj-docx-1-dhj-10.1177_20552076251362753 - Supplemental material for Poisoning accidents in young children—Theory-based evaluation of an mHealth app Supplemental material, sj-docx-1-dhj-10.1177_20552076251362753 for Poisoning accidents in young children—Theory-based evaluation of an mHealth app by Patricia Müller, Annett Schulze, Johanna Geppert, Axel Menning, Fabian Brand, Paula Stehr, Doreen Reifegerste and Constanze Rossmann in DIGITAL HEALTH

sj-docx-2-dhj-10.1177_20552076251362753 - Supplemental material for Poisoning accidents in young children—Theory-based evaluation of an mHealth app Supplemental material, sj-docx-2-dhj-10.1177_20552076251362753 for Poisoning accidents in young children—Theory-based evaluation of an mHealth app by Patricia Müller, Annett Schulze, Johanna Geppert, Axel Menning, Fabian Brand, Paula Stehr, Doreen Reifegerste and Constanze Rossmann in DIGITAL HEALTH
