# Supplementary material for: Poisoning accidents in young children—Theory-based evaluation of an mHealth app
Source: Digit Health. 2025 Aug 6;11:20552076251362753. doi: 10.1177/20552076251362753 (PMC12329195; doi:10.1177/20552076251362753)
Supplement: sj-docx-1-dhj-10.1177_20552076251362753 - Supplemental material for Poisoning accidents in young children—Theory-based evaluation of an mHealth app [file sj-docx-1-dhj-10.1177_20552076251362753.docx]

# COREQ-Checklist Poisoning Accidents in Young Children – Theory-Based Evaluation of an mHealth App

In this overview we answer all questions required by the COREQ-protocol for reporting qualitative studies.

Appendix 1: Consolidated criteria for reporting qualitative research (COREQ)

| No. and topic | | | Guide questions/description | Reported on | Page Nr. |
| --- | --- | --- | --- | --- | --- |
| Domain 1: Research team and reflexivity | | | | |  |
| *Personal Characteristics* | | | | |  |
| 1 | Interviewer/facilitator | Which author/s conducted the interview or focus group? | | Data collection | 8 |
| 2 | Credentials | What were the researcher’s credentials? *(e.g., PhD, MD)* | | Design | 5 |
| 3 | Occupation | What was their occupation at the time of the study? | | Data collection | 5, 8 |
| 4 | Gender | Was the researcher male or female? | | Data collection | 8 |
| 5 | Experience and training | What experience or training did the researcher have? | | Design | 5 |
| *Relationship with participants* | | | | |  |
| 6 | Relationship established | Was a relationship established prior to study commencement? | | Data collection | 8 |
| 7 | Participant knowledge of the interviewer | What did the participants know about the researcher? *(e.g., personal goals, reasons for doing the research?)* | | Data collection | 8 |
| 8 | Interviewer characteristics | What characteristics were reported about the interviewer/facilitator? *(e.g., bias, assumptions, reasons and interest in the research topic?)* | | Data collection | 8 |
| Domain 2: study design | | | | |  |
| *Theoretical framework* | | | | |  |
| 9 | Methodological orientation and Theory | What methodological orientation was stated to underpin the study? *(e.g., grounded theory, discourse analysis, ethnography, phenomenology, content analysis)* | | Data analysis | 11 |
| *Participant selection* | | | | |  |
| 10 | Sampling | How were participants selected? *(e.g., purposive, convenience, consecutive, snowball)* | | Participant selection | 6-7 |
| 11 | Method of approach | How were participants approached? *(e.g., face-to-face, telephone, mail, e-mail)* | | Participant selection | 7 |
| 12 | Sample Size | How many participants were in the study? | | Study participants | 11-12 |
| 13 | Non-participation | How many people refused to participate or dropped out? Reasons? | | Study participants | 11 |
| *Setting* | | | | |  |
| 14 | Setting of data collection | Where was the data collected? *(e.g., home, clinic, workplace)* | | Data collection | 7-8 |
| 15 | Presence of non-participants | Was anyone else present besides the participants and researchers? | | Data collection | 7-8 |
| 16 | Description of sample | What are the important characteristics of the sample? *(e.g., demographic data)* | | Study participants | 11-12 |
| *Data Collection* | | | | |  |
| 17 | Interview guide | Were questions, prompts, guides provided by the authors? Was it pilot tested? | | Data collection | 9-10 |
| 18 | Repeat interviews | Were repeat interviews carried out? If yes, how many? | | n/a |  |
| 19 | Audio/visual recording | Did the research use audio or visual recording to collect the data? | | Data collection | 8 |
| 20 | Field notes | Were field notes made during and/or after the interview or focus group? | | Data collection | 8 |
| 21 | Duration | What was the duration of the interviews or focus group? | | Data collection | 10 |
| 22 | Data saturation | Was data saturation discussed? | | Participant selection | 7 |
| 23 | Transcripts returned | Were transcripts returned to participants for comment and/or correction? | | n/a |  |
| Domain 3: analysis and findings | | | | |  |
| *Data analysis* | | | | |  |
| 24 | Number of data coders | How many data coders coded the data? | | Data analysis | 11 |
| 25 | Description of the coding tree | Did authors provide a description of the coding tree? | | Results (Figure 3) | 13 |
| 26 | Derivation of themes | Were themes identified in advance or derived from the data? | | Data analysis | 11 |
| 27 | Software | What software, if applicable, was used to manage the data? | | Data analysis | 11 |
| 28 | Participant checking | Did participants provide feedback on the findings? | | n/a |  |
| *Reporting* | | | | |  |
| 29 | Quotations presented | Were participant quotations presented to illustrate the themes / findings? Was each quotation identified? *(e.g., participant number)* | | Results | 13-19 |
| 30 | Data and findings consistent | Was there consistency between the data presented and the findings? | | Results | 13-19 |
| 31 | Clarity of major themes | Were major themes clearly presented in the findings? | | Results | 13-19 |
| 32 | Clarity of minor themes | Is there a description of diverse cases or discussion of minor themes? | | Results | 13-19 |
