# Supplementary material for: Poisoning accidents in young children—Theory-based evaluation of an mHealth app
Source: Digit Health. 2025 Aug 6;11:20552076251362753. doi: 10.1177/20552076251362753 (PMC12329195; doi:10.1177/20552076251362753)
Supplement: sj-docx-2-dhj-10.1177_20552076251362753 - Supplemental material for Poisoning accidents in young children—Theory-based evaluation of an mHealth app [file sj-docx-2-dhj-10.1177_20552076251362753.docx]

# Interview guide

# Poisoning Accidents in Young Children – Theory-Based Evaluation of an mHealth App

## General introduction

Introducing oneself as interviewer.

Like the scenario session, the interview is also recorded and anonymized.

Please feel free to ask if a question seems unclear to you.

And you can continue to share the app with us on the screen, just as you did in the scenario session. You can access the app at any time to provide answers to my question.

You have already completed seven tasks with the BfR app now and have gained an impression of how the app is structured and how the app can be navigated.

| **Topic** | **Introduction and questions** | **Follow-up questions** |
| --- | --- | --- |
| Perceived usefulness  *Use in daily life* | **Introduction**  You have dealt with very different scenarios:   - “Moving into a new flat and childproof the home” - “Assess the risks of cosmetics and products used in the bathroom” - “Discovering a new plant in the garden” - “Kid having swallowed a paperclip” - “Putting together a family medicine cabinet” - “Kid having swallowed a tablet of Ramipril” - “Discovering cleaning agent residues on your child's face and contact PCC”. |  |
|  | **Questions**   1. In which scenario did you feel that you made good progress in managing the scenario? What was the reason for this? 2. Apart from what you have just told me, are there any other tasks that you were particularly good at? 3. And vice versa: Was there a scenario that was particularly challenging for you? What was the reason for this? | Why was it easy? |
|  | 1. And if you now think about your daily life: Can you imagine using this app in your everyday life?    1. Why? Why rather not?    2. If necessary, follow up: Which parts of the app are helpful for your child's safety?    3. Which functions do you find particularly helpful?    4. What information is helpful?    5. What would have to be different to offer you more safety? What did you miss? |  |
| Perceived ease of use | 1. Generally speaking, how easy or difficult did you find it to use the app? What helped you to use it? Where did problems arise?    1. How did you like the design of the app?       1. Was there specific content in the app that you were able to understand particularly well? What do you think was the reason for this?       2. What content did you find challenging? In how far?   **Introduction**  Let’s go back to scenario 2, in which you were asked to classify the dangers and risks of various care and cosmetic products:  **Questions**   - 1. How well or not so well were you able to search for information? (Expertise in searching for information)   2. Let’s take a look at the start screen together:      - 1. What do you associate with these symbols? Did what was meant by this meet your expectations? (Familiarity with symbols/with the user interface)        2. Were you familiar with the terms? (Familiarity with terms) | Why helpful?  For example? Where exactly in the app?  Referencing scenario 2 |
| Recommendations | **Introduction**  In the last couple of minutes, we have mainly talked about aspects of the app that you like and about challenges in the app. If you think about potential improvements now:  **Questions**   1. How could the app be improved?    1. Looking at the navigation menu?    2. Looking at the design of the app?    3. Looking at the design of the content? (Photos, text elements etc.) |  |
| Trust  *Trust*  *Situations in which the app could be used*  *App publisher*  *Privacy and trust*  *Keeping the app installed* | **Introduction**  Now we get to the final part of the interview, which is about trust.  **Questions**   1. Would you trust the app for preventing poisoning accidents in children as a source of advice?    1. If necessary: In how far would you trust this app as an advisor?    2. If necessary: In which situations would an app like the BfR app “Poisoning accidents in children” be beneficial?    3. If necessary: Please think of task 6, the swallowing of a medicine, and task 7, the discovery of cleaning agent residues on the face of the child you take care of.   Would you use the app in a critical situation like this? Why/why not?   1. Is it important to you who created this application? In how far?   **Introduction**  When you think about protecting your privacy/data protection:  **Questions**   1. Did you feel safe using the app? Please explain why. 2. Would you keep the app installed on your smartphone?   If necessary:   - 1. Would you use the app for prevention?   2. Would you use the app in an emergency? | What are the aspects that trigger trust?  Why was that – referring to individual data. |
| Conclusion | **Introduction**  Finally, I would like to ask you if there is anything else that we haven't talked about but that is important to you and that you would like to tell me? |  |
| Farewell | Then we have now reached the end of the interview. Thank you for your time.  Please finish sharing your screen with your smartphone first. You can now leave the Zoom meeting. Simply click on the “End” button. |  |
